# Solid-state NMR chemical shift analysis for determining the conformation of ATP bound to Na,K-ATPase in its native membrane†

**DOI:** 10.1039/d3ra06236h

**Published:** 2023-11-29

**Authors:** David A. Middleton, John Griffin, Mikael Esmann, Natalya U. Fedosova

**Affiliations:** a Department of Chemistry, Lancaster University Bailrigg Lancaster LA1 4YB UK d.middleton@lancaster.ac.uk +44 (0)1524 594328; b Department of Biomedicine, Aarhus University Aarhus Denmark

## Abstract

Structures of membrane proteins determined by X-ray crystallography and, increasingly, by cryo-electron microscopy often fail to resolve the structural details of unstable or reactive small molecular ligands in their physiological sites. This work demonstrates that ^13^C chemical shifts measured by magic-angle spinning (MAS) solid-state NMR (SSNMR) provide unique information on the conformation of a labile ligand in the physiological site of a functional protein in its native membrane, by exploiting freeze-trapping to stabilise the complex. We examine the ribose conformation of ATP in a high affinity complex with Na,K-ATPase (NKA), an enzyme that rapidly hydrolyses ATP to ADP and inorganic phosphate under physiological conditions. The ^13^C SSNMR spectrum of the frozen complex exhibits peaks from all ATP ribose carbon sites and some adenine base carbons. Comparison of experimental chemical shifts with density functional theory (DFT) calculations of ATP in different conformations and protein environments reveals that the ATP ribose ring adopts an C3′-*endo* (N) conformation when bound with high affinity to NKA in the E_1_Na state, in contrast to the C2′-*endo* (S) ribose conformations of ATP bound to the E2P state and AMPPCP in the E1 complex. Additional dipolar coupling-mediated measurements of H–C–C–H torsional angles are used to eliminate possible relative orientations of the ribose and adenine rings. The utilization of chemical shifts to determine membrane protein ligand conformations has been underexploited to date and here we demonstrate this approach to be a powerful tool for resolving the fine details of ligand–protein interactions.

## Abstract 2

Solid-state NMR and DFT ^13^C chemical shift calculations are used to determine the ribose ring conformation of hydrolysable adenosine 5′-triphosphate when freeze-trapped in the high-affinity binding site of Na,K-ATPase.

## Graphical abstract

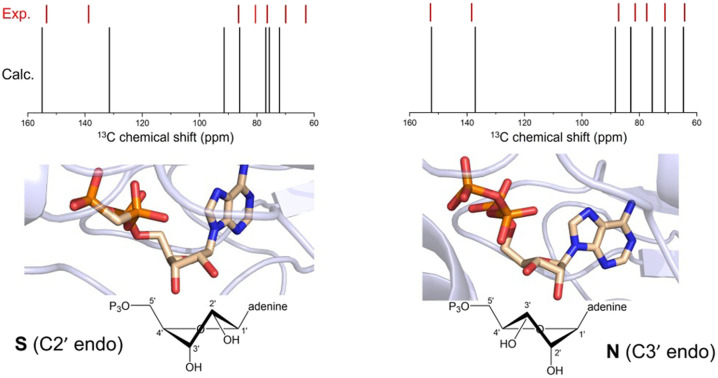

## Introduction

Cell membrane-embedded proteins are encoded by approximately 30% of mammalian genomes and constitute over half of pharmaceutical targets.^1^ The last 20 years have borne witness to an exponential increase in the number of published atomic-level structures of membrane proteins (see, *e.g.*, https://blanco.biomol.uci.edu/mpstruc/), determined predominantly by X-ray crystallography, but increasingly by cryo-electron microscopy.^2^ It is important to visualise within these structures the binding sites and conformations of biological ligand molecules in order to fully understand the regulation of protein function and to inform drug design.^3^ Unfortunately, many membrane protein structures lack these fine details because of the challenges of maintaining stable ligand–protein complexes, particularly when the ligand is a reactive enzyme substrate.

Magic-angle spinning (MAS) solid-state NMR (SSNMR) spectroscopy is a valuable tool for observing reactive ligands, and stable analogues thereof, in the binding sites of membrane proteins in native or reconstituted lipid bilayers.^4–6^ SSNMR restraints on the molecular conformations of membrane protein ligands have historically been derived from internuclear distance-dependent dipolar coupling measurements.^7–9^ Chemical shifts also carry a wealth of information about molecular conformation, environment and interactions, but have been under-utilised in the structural analysis of membrane protein ligands by SSNMR. Computational quantum chemical (*e.g.*, DFT) calculations to analyse experimentally-determined chemical shifts are now used widely, including for analysing the structures of water-soluble ligand–protein complexes from solution and solid-state chemical shifts.^10–12^ Simulations using atom-centred Gaussian basis sets correlate extremely well with experimental data, and can be computationally inexpensive if focused clusters of ligand and protein atoms are used judiciously to approximate the larger system.^13^ For periodic systems including crystalline molecules, plane wave basis sets are applied with pseudopotentials replacing the core electrons.^14–16^ Both approaches require the comparison of sufficient calculated and experimental chemical shift data to answer a particular structural question. SSNMR detection of membrane protein-bound ligands for chemical shift analysis requires costly and often challenging isotope (*e.g.*, ^13^C) enrichment for sensitivity enhancement, which has limited the scope of chemical shift analysis in this context.

Adenosine 5′-triphosphate (ATP) is a ubiquitous molecular energy source, the hydrolysis of which drives many biological processes and actively maintains ionic homeostasis across living cells. MAS SSNMR has been used to examine ATP interactions with membrane-embedded proteins by detecting signals from the intrinsic ^31^P nuclei.^17^ Rates of ATP hydrolysis by the ABC multidrug transporter, LmrA, in reconstituted membranes at 290 K were measured by real-time detection of the emerging ^31^P_β_ signal from ADP.^18^^31^P SSNMR of another multidrug transporter, BmrA, revealed that ATP binds strongly to two binding sites, whereas a trapped transition state intermediate binds strongly to only one site and the hydrolytic product, ADP, binds weakly to both sites.^19^ The non-hydrolysable ATP analogues AMPPCP and ADP·AlF_4_^−^, which is used to model intermediate states, are useful for SSNMR studies on sedimented membranes at non-freezing temperatures.^6^ Alternatively, hydrolysis of authentic ATP can be prevented by freeze-trapping the nucleotide in its native binding site.^4,5^ Importantly, ATP can be readily prepared with uniform ^13^C enrichment, which allows for an extensive structural investigation by chemical shift analysis.

Here, SSNMR measurements and DFT chemical shift calculations are performed to obtain new details on the molecular conformation of [U–^13^C]ATP when bound to Na,K-ATPase (NKA). NKA is a member of the P-type ATPase family that utilizes the free energy of ATP hydrolysis to exchange intracellular Na^+^ for K^+^. Ion exchange is facilitated by two enzyme conformations called E1 (high-affinity for Na^+^ and ATP) and E2 (high-affinity for K^+^), *via* intermediate phosphorylated states (E1P and E2P).^20^ Several structures of NKA are available that resolve bound ATP and its non-hydrolysable proxies, but the structure of the native ATP substrate when bound to NKA in the high-affinity E1 state is unknown. This is because, when sufficient Na^+^ is present to induce the E1-conformation, NKA exhibits high affinity towards ATP and hydrolyses the nucleotide to ADP + Pi, rapidly in the presence of Mg^2+^ and at a slower rate in the absence of Mg^2+^.^21^ Inspection of the available structures reveal that ATP and its non-hydrolysable proxies do not adopt a unique structure when bound to the enzyme, and the ribose ring conformation is variable (ESI, Fig. S1 and S2†).

We analyse the ^13^C chemical shifts for ATP bound to the E1 form of NKA, preserved from hydrolysis by freeze-trapping SSNMR, to uncover detailed information about the ribose sugar conformation of the native, hydrolysable nucleotide in the active site.

## Materials and methods

### Sample preparation

NKA membranes from shark rectal gland were prepared and complexed with [U–^13^C]ATP–NKA as described in detail previously.^5,22^ Briefly, membranes were sedimented by ultracentrifugation and homogenized with [U–^13^C]ATP in a small volume (10 μL) of buffer. The hydrated sedimented pellet was transferred to a 4 mm diameter magic-angle spinning sample rotor and flash-frozen in liquid nitrogen less than 10 minutes after the addition of ATP. The sample was maintained at −25 °C or lower thereafter, including during the NMR measurements.

### NMR measurements

Spectra were obtained on a Bruker Avance 400 spectrometer operating at a Larmor frequency of 100.13 MHz for ^13^C. The temperature was maintained at −25 °C for the duration of each experiment. Membrane samples were spun at a MAS rate of 8 kHz and standard 1D CP-MAS NMR spectra were obtained by averaging 170 000 transients. Experimental parameters were: a 4.0 μs ^1^H excitation pulse, 2 ms Hartmann–Hahn cross polarization at 65 kHz for ^1^H and 90 kHz for ^13^C, two-pulse phase modulated (TPPM) proton decoupling^23^ at a field of 85 kHz during signal acquisition and a 2 s recycle delay pulse. The selective HCCH experiment employed the pulse sequence given in Fig. S3.† Experimental parameters were: MAS at 5226 kHz, a 4 μs ^13^C π/2 pulse, 1.5 ms zero-quantum excitation time and Lee-Goldberg proton homonuclear decoupling during the dipolar evolution period of one rotor cycle.

### Computational approach for DFT calculations

The approach for the computational calculations adopted here followed on from an earlier, unrelated DFT analysis of microcrystalline ATP chemical shifts measured by SSNMR (not presented). In the previous study, chemical shift calculations were performed by plane-wave DFT using the gauge-including projector augmented wave (GIPAW) algorithm,^24^ which allows the reconstruction of the all-electron wave function in the presence of a magnetic field. All procedures are implemented in the Cambridge Serial Total Energy Package (CASTEP).^25^ This approach provides accurate approximations of large systems by applying periodic boundary conditions to crystal unit cells used as simulation boxes and propagating the calculated cell properties in the chosen dimensions.

For convenience, it was decided to also apply the CASTEP methodology here to calculate ^13^C chemical shifts for protein-bound ATP in order to distinguish between the possible ribose ring conformations of ATP in the high-affinity nucleotide site of NKA. Although the plane wave-GIPAW approach is designed for three-dimensional periodic arrays, calculations can be performed on any type of system, including single molecules and non-repeating extended clusters of atoms.^26^ The calculations incorporate contributions to the observed shifts from intramolecular and intermolecular factors. It should be noted, however, that the assumption of periodic boundary conditions is not necessary for the system investigated, and that there is trade-off between the additional computation time of the plane wave-GIPAW approach and the accuracy of the functional employed. A generalized gradient approximation (GGA) functional (PBE) was used to minimise calculation time. It will be shown that this method is sufficient to distinguish between the two principal ribose conformations of ATP when compared with chemical shifts and the attendant errors imposed by the measured line widths and signal to noise. More accurate calculations can in principle be achieved in similar or shorter time frames using Gaussian with hybrid functionals (*e.g.*, B3LYP). These were not considered to be superior to the CASTEP approach here, because of the experimental errors imposed by the measured line widths and signal to noise.

### Model building for DFT calculations

For calculations on isolated ATP molecules, the coordinates of ATP were extracted from the appropriate PDB file and a pseudo-triclinic (*P*1) unit cell of dimensions 25 × 25 × 20 Å was set up as the simulation box, using the software CrystalMaker®. The atomic positions were expressed as fractional coordinates in this unit cell. For calculations on ATP in the NKA binding site, a simulation box of 30 × 30 × 30 Å was used to contain the coordinates of ATP and selected binding residues from NKA. The simulation box comprised 220–250 atoms, depending on which structure and form of NKA was used. The main chain of each amino acid residue in the box was capped with NH_2_ and CONH_2_ groups at the N- and C-termini, respectively, to saturate the bonds. All side chains were protonated so as to sustain no net charge.

The molecular geometry within the simulation box was optimised within the CASTEP environment. All atomic positions were allowed to vary and the Grimme G06 semi-empirical dispersion correction scheme was used.^27^ Typical optimisation times for a 230 atom system on a high-end computer with 4 parallel nodes were 48–52 hours. Additionally, some optimisations fixed the heavy atoms and only the hydrogen atoms were varied. In these cases, typical optimisation times were reduced to 26–30 hours. An optimisation was considered acceptable if the RMSD of the new and old atomic coordinates was less than the resolution of the crystal structure from which the original coordinates were extracted.

### Chemical shift calculations

The CASTEP DFT calculations employed the GGA PBE functional^28^ and core–valence interactions were described by ultrasoft pseudopotentials.^27^ Calculations were performed using a planewave energy cut-off of 50 Ry (680 eV) and due to the large cell size, a single *k*-point at the fractional coordinate (0.25, 0.25, 0.25) in reciprocal space for integration over the Brillouin zone. The outcome of the calculations include the isotropic ^13^C magnetic shielding values for ATP ribose and adenine moieties. These values were converted to chemical shifts (in ppm) by subtracting them from the calculated shielding values for a reference molecule, adamantane.^29,30^ Water molecules were not included in these calculations, but in separate calculations on isolated ATP structures in which water molecules were placed at hydrogen bonding positions relative to the ribose ring OH groups did not have a major effect on the calculated ^13^C chemical shifts. Calculations took 1–3 hours depending on the number of atoms in the system (47 for isolated ATP, 230–250 for binding clusters).

### Computational simulations of the HCCH data

HCCH experiments monitor the evolution of ^13^C–^13^C double quantum coherence over one cycle of sample rotation at the magic-angle, and is influenced by the relative orientations of the C–H bonds. Simulations of DQ evolution under ^1^H dipolar coupling measured in a dipolar-chemical shift (DIPSHIFT) SSNMR experiment were undertaken to find the values of dynamically-averaged dipolar coupling constants providing the closest fit to the data. Simulations were carried out in the SIMPSON software environment.^31^ The simulation variables were: the MAS rate, H–C–C bond angles, H–C–C–H torsional angle and the ^1^H–^13^C dipolar coupling constants multiplied by a scaling factor determined experimentally on a standard. All variables with the exception of the torsional angle were taken directly, or calculated, from the molecular geometry of ATP, averaged across all molecular structures examined in this work.

## Results and discussion

### Conformational preferences of the ATP ribose in diverse protein binding sites

**Fig. 1:**
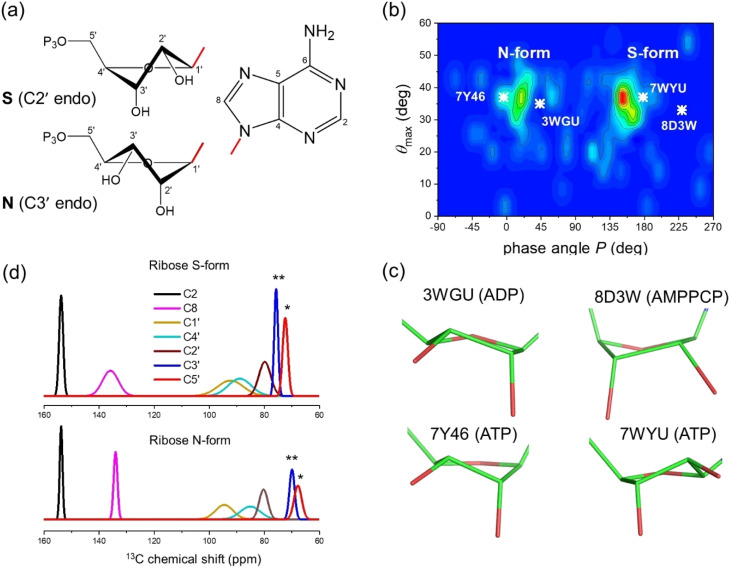
Analysis of ribose conformations of ATP molecules bound to proteins with structures deposited in the PDB. (a) The two principal ribose conformations identified in nucleotides and nucleosides. (b) Distribution of ATP ribose ring conformations in 272 PDB files of protein structures. Conformations are represented by pseudorotation factor *P* and maximum amplitude *θ*_max_. The white crosses denote the ribose conformations of ATP in: the cryo-EM structure of NKA in the E2P state formed in the presence of ATP (PDB 7WYU);^39^ the E2.2K^+^ state after addition of ATP (PDB 7Y46);^38^ the E1·Na^+^ state with ADP and AlF_4_^−^ (PDB 3WGU);^41^ and the E1 state with AMPPCP (PDB 8D3W).^42^ (c) Ribose ring conformations of ATP, AMPPCP and ADP in the 4 NKA structures highlighted in (b). (d) Plane wave-GIPAW DFT calculations (with the GGA functional) of ^13^C isotropic chemical shifts for ATP ribose carbons. Shift distributions calculated from ATP atomic coordinates (10 ribose S-form and 10 ribose N-form) isolated from 20 PDB protein structures. Normal distributions are assumed and based on the calculated means and standard deviations, *σ*, given in Table 1. Asterisks denote significant differences between C3′ at the *p* < 0.05 (*) and for C5′ at the *p* < 0.01 levels (**).

The ^13^C chemical shifts of 5- and 6-membered carbohydrate rings, including riboses, are sensitive to their conformation, environment and interactions.^32–34^ Pseudorotation of ribose rings in RNA nucleotides results in two major conformations, referred to as the S (or C2′-*endo*) and N (or C3′-*endo*) forms (Fig. 1a). The ring conformation can be described by two parameters, the angle of pseudorotation, *P*, and the degree of pucker, *θ*_max_, defined as:^35,36^1
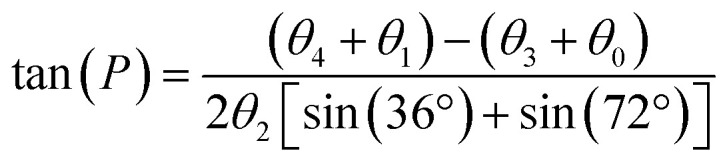
2
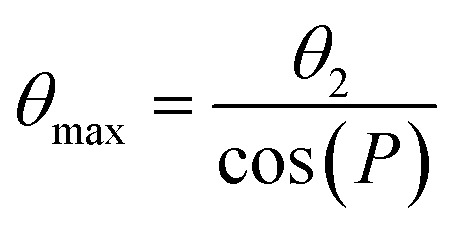
where *θ*_*n*_ are the torsional angles defining the ribose ring conformation. Suardiaz *et al.* applied DFT calculations to a database of ribose structures in RNA to derive an equation relating the ribose structural parameters and the ^13^C chemical shifts.^37^ Good empirical correlations have been established between the ribose C1′ shift and the base orientation for the deoxyribose group, confirming DFT calculations.^36^Fig. 1b illustrates the conformational variability of the ATP ribose ring in 272 diverse and unrelated protein structures taken from the PDB. The ATP ribose ring conformations fall predominantly into the two forms, S and N, in approximately equal proportions, with approximately 10% in outlying conformations.

The only structures of hydrolysable ATP when bound to NKA are of the enzyme in the phosphorylated E2P conformation (PDB 7WYU; 3.4 Å)^38^ and potassium-bound E2.2K^+^ conformation (PDB 7Y46; 7.2 Å),^39^ which both have low affinity for ATP and do not hydrolyse the nucleotide. Crystal structures have been determined for NKA in the E1·Na^+^ state in the presence of the ADP·AlF_4_^−^ proxy to trap the enzyme in the transition state preceding E1P (PDB 4HQJ and 3WGU; resolution 4.3 Å and 2.8 Å),^40,41^ and in the E1 state with the AMPPCP proxy (PDB 8D3W; 3.5 Å).^42^ Inspection of the structures of ATP and its analogues in these binding sites indicates that the ribose group adopts N-type conformations in 3WGU and 7Y46 (Fig. 1b and c, left) and S-type conformations in 7WYU and 8D3W (Fig. 1b and c, right). The conformation of NKA, E1 or E2, does not appear therefore to influence the conformation of the ATP ribose ring.

### Calculation of ^13^C chemical shifts for protein-bound ATP in N- and S-ribose forms

Quantum chemical calculations of ^13^C chemical shifts were carried out to determine how the ribose conformations of protein-bound ATP molecules influence the ribose and adenine ring ^13^C chemical shifts. Coordinates of ATP molecules classified as either S- or N-ribose forms were extracted from the 272 diverse protein structure files represented in Fig. 1b. The ATP molecules were each confined within a simulation box of 25 × 25 × 20 Å representing a fictitious unit cell and, after structural optimisation, the ^13^C chemical shifts were calculated for each molecule in isolation. The unit cell size was optimised to eliminate nearest neighbour contributions to the calculated shifts. After all-atom geometry optimisation, only structures with new coordinates within an acceptable RMSD of the original positions were accepted. A total of 20 acceptable structures were used in the chemical shift calculations (10 molecules for each form).

Fig. 1d illustrates the distribution of ^13^C chemical shifts calculated for the ribose and adenine carbons of the isolated ATP molecules in the two forms, when bound to the sample of 20 proteins. A normal distribution is assumed about the mean chemical shift, in which the distribution width for each carbon represents ±2*σ*. The distribution widths for the ribose ^13^C chemical shifts reflect their sensitivity to the range of ring conformations within the N- and S-groups, as expected. It is surprising that the distribution of shifts for C8 is rather broad for the ribose S-form, considering that the adenine ring is planar and conformationally invariant, and suggests that the ribose ring may have a long-range influence on the shielding at this carbon. The ^13^C chemical shift distributions for the ribose C1′, C2′ and C4′ carbons are broad and differences between the ribose S and N forms are statistically insignificant, but the shifts for C3′ and C5′ are statistically different. This observation agrees with earlier conclusions that the chemical shielding of the C3′ carbon is the most sensitive to the sugar ring pucker, with a variation of up to 10 ppm between the C3′ *endo* and C2′ *endo* conformations.^34^

### ^13^C chemical shift measurements for ATP in the high-affinity site of NKA

**Fig. 2:**
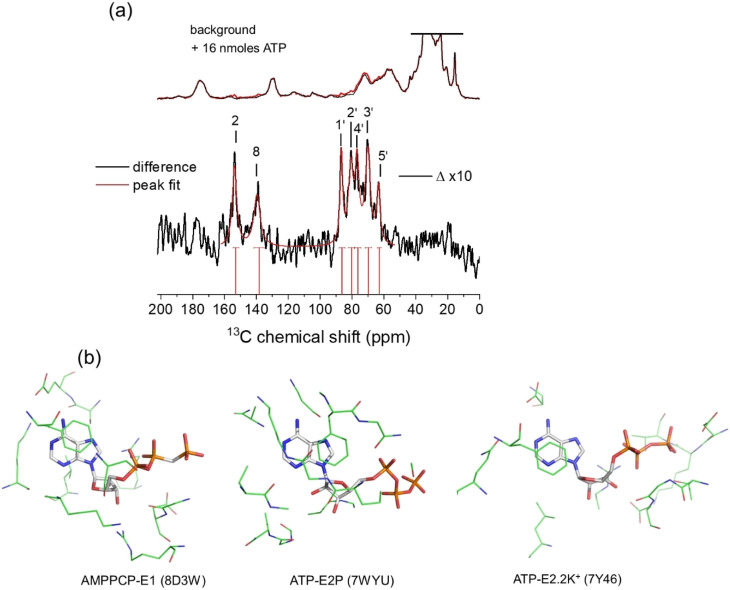
(a) ^13^C CP-MAS SSNMR spectra of shark membrane preparation of NKA in the E1·Na^+^ state alone (top black) and containing 16 nmoles [U–^13^C]ATP (red) at −25 °C. The difference spectrum (*Δ*), with assignments, obtained by subtraction of the background spectrum is shown 10-fold vertically expanded. Spectra were the results of accumulating 170 000 transients with an experiment time of 94 h per spectrum. The combined Lorentzian line of best fit to the 7 observable peaks is overlaid in red. Vertical red lines and horizontal error bars represent the mean and probable errors in the chemical shifts, as described in Table 1. (b) Structures of AMPPCP and ATP in the binding sites of NKA in E1 and E2 conformations. In each case, the nucleotide and residues in closest contact shown represent the fictional unit cells (volume 27 × 10^3^ Å^3^) used as the simulation box in the DFT calculations of nucleotide ^13^C chemical shifts.

The ^13^C chemical shifts for authentic ATP when bound to NKA in the E1 state were next measured in order to determine the ribose conformation and orientation. SSNMR has previously detected signals from uniformly ^13^C-labelled ATP when freeze-trapped at −25 °C in the high-affinity nucleotide site of NKA in the E1 form.^4,5,43^Fig. 2a shows ^13^C cross-polarization magic-angle spinning (CP-MAS) SSNMR spectra (at a proton Larmor frequency of 400.13 MHz) of fully-functional membrane preparations of NKA, with Na^+^ added to induce the E1 conformation. Spectra are shown for membranes prepared in the absence of nucleotide and with NKA complexed with [U–^13^C]ATP and freeze-trapped at −25 °C. The NKA-nucleotide complex was prepared such that over 90% of the ATP present (approximately 16 nmoles) binds to the high-affinity site.^5,22^ Clear resonances from all the CH and CH_2_ carbons are observed in a difference spectrum after subtraction of the natural abundance signals. Resonances from non-protonated carbons are not observed. This is because the Hartmann–Hahn contact time (2 ms) was optimised on microcrystalline ATP to maximise the signals from CH carbons of the ribose ring (and adenine carbons C2 and C8). Longer contact times are required to observe non-protonated carbons above the noise, because the rate of cross-polarization from more weakly coupled neighbouring protons is slower than it is between directly bonded C–H spins. The MAS rate was sufficiently high (8 kHz) that the observed peak frequencies for ATP are virtually identical to the isotropic values, as confirmed with simulated spectra based on known ^13^C chemical shift anisotropies.^44^ The effect of the ribose ^13^C anisotropies are largely removed at 8 kHz MAS and the peak intensities would not increase significantly at higher MAS rates.

The peaks for the ribose carbons and adenine C2 and C8 are considerably broader (∼3–4 ppm) than typically seen in solution-state spectra at room temperature (<0.5 ppm). The increased widths may be attributed to ^13^C–^13^C *J*-coupling, the structural heterogeneity of ATP and its local environment and to the line broadening applied to improve signal-to-noise. Consequently, the chemical shifts for each site are specified as ranges defined by the peak widths, with the mean values at the peak maxima (Table 1). The mean shifts are notably different from those for [U–^13^C]ATP in aqueous solution. These differences arise because free and bound ATP adopt distinct molecular conformations, or because of differences in the local environment of the ^13^C nuclei in the free and bound states, or (most likely) a combination of the two effects. Interestingly, the chemical shifts for C3′ and C5′ fall within the range of values calculated for the isolated ATP molecules in the N-form (Fig. 1d and Table 1). However, conclusions about the ribose ring conformation cannot be drawn until the contribution of the binding environment to the observed ^13^C chemical shifts is known. For example, carbohydrate ring NMR shift parameters can be sensitive to C–H⋯O hydrogen bonding lengths and angles in crystalline solids, although ^13^C shifts are less sensitive than ^1^H shifts to the exact hydrogen-bonding arrangement.^45,46^

**Table 1:** Summary of experimental and calculated ^13^C chemical shifts for ATP in different environments. All chemical shifts are referenced to adamantane

ATP environment	Chemical shift *δ*_i_ (ppm)						
	C1′	C2′	C3′	C4′	C5′	C2	C8

**Experimental**							
NKA E_1_^a^	86.8 (3.6)	76.7 (3.0)	70.1 (3.9)	80.4 (3.6)	63.2 (2.5)	153.5 (3.3)	139.0 (4.8)
Aqueous^b^	89.9 (1.2)	77.0 (1.2)	72.3 (1.3)	86.9 (1.0)	67.6 (1.0)	155.2 (1.2)	142.2 (1.3)
Aqueous^c^	89.5	77.0	72.9	86.6	67.9	155.4	142.5

**Calculated**							
S-Form^c^	92.3 (4.7)	79.9 (2.1)	75.7 (0.7)	88.9 (4.2)	72.4 (0.9)	153.8 (0.7)	135.9 (2.9)
N-Form^d^	94.6 (3.2)	80.3 (1.6)	69.9 (0.9)	85.1 (3.6)	67.8 (1.4)	153.8 (0.5)	134.0 (0.7)
7WYU isolated	96.2	80.8	75.7	89.1	73.5	152.8	136.1
7WYU bound	95.9	80.8	76.6	88.1	72.6	152.4	134.9
7Y46 isolated	88.9	77.9	69.5	86.5	63.9	152.6	134.8
7Y46 bound	88.4	75.6	71.0	83.0	64.7	151.4	135.2
8D3W isolated	91.4	76.9	75.6	86.0	72.1	154.9	131.5
8D3W bound	91.0	74.6	76.9	85.6	72.9	153.8	131.9

a Mean chemical shifts (peak maxima) and full widths at half height (FWHH) measured by Lorentzian curve fitting to the difference spectrum in Fig. 2a. The FWHH values (given in brackets) represent the probable errors in the measured chemical shifts according to the Cauchy distribution.

b From a direct polarization MAS NMR spectrum of frozen aqueous solution of 1 mM [^13^C]ATP at −25 °C.

c Values taken from the Biological Magnetic Resonance Data Bank.

d Means (*σ*) calculated from 10 protein structure files from the PDB.

### Chemical shift calculations for ATP bound to NKA

Next ^13^C chemical shift calculations were performed to determine how the combined effect of the NKA binding environment and nucleotide molecular conformation influences the ^13^C chemical shifts. Calculations were performed on ATP and AMPPCP coordinates isolated from their respective crystal structures, and again after reintroducing the key binding residues. We focused on the structures of ATP bound to NKA in the E2.2K^+^ state (PDB 7Y46), in which the ribose moiety adopts the N-form, and on the structures of ATP bound to the E2P state (PDB 7WYU) and of AMPPCP bound to the E1 state (PDB 8D3W), which both carry the S-form ribose.

Calculations were set up with a simulation box of dimension 30 × 30 × 30 Å containing ATP (or AMPPCP) alone or in a cluster with selected binding residues (Fig. 2b). Clusters of residues around the ATP binding site of NKA in the E2P and E2.2K^+^ states were Phe-482, Arg-551, Leu-553, Lys-487, Asp-619 and Gly-618. For AMPPCP bound to NKA in the E1 state, the selected residues were Ser-452, Glu-453, Phe-482, Ser-484, Lys-487, Lys-508, Arg-551, Leu-553, Asp-619 and Arg-692. These residues collectively participate in hydrogen bonding, van der Waals and π–π interactions with the bound ligand, potentially affecting the observed chemical shifts. The number of atoms was restricted to those situated ≤4 Å from ATP (230–250 atoms including ATP) and having greatest influence on chemical shifts, so as to maintain a practicable computational time (which scales as the cube of the system size).

**Fig. 3:**
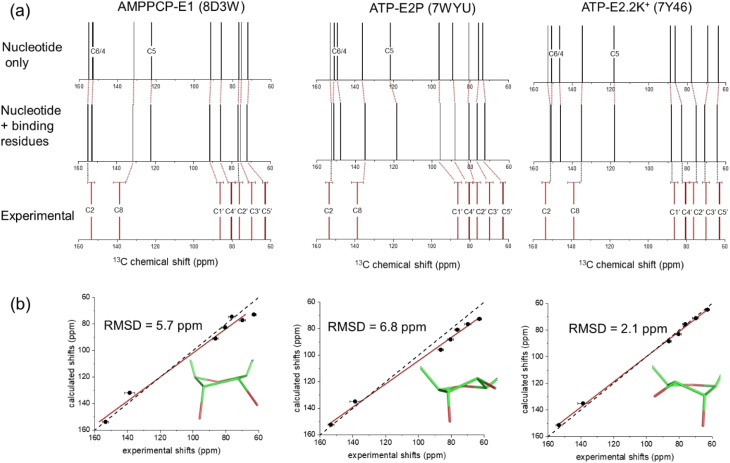
DFT calculations of ^13^C isotropic chemical shifts for all ribose and selected adenine carbons of ATP and AMPPCP bound to NKA in E1 and E2 conformations. (a) Calculated shifts for the nucleotide in the binding conformation after isolation from all protein residues (top), calculated shifts for the nucleotide after reintroducing the binding site residues in closest proximity (within 4 Å) to the nucleotide, and experimental chemical shift values for ATP bound to NKA in the E1 conformation (bottom). (b) Correlation of experimental chemical shifts and calculated values for nucleotide in the presence of binding residues. The line of best fit (red) is shown together with the line representing *y* = *x* (black). The carbon numbering scheme is given in Fig. 1a. Error bars in panels (a) and (b) represent the FWHH, as described in Table 1.

Fig. 3a shows the chemical shifts calculated for ATP and AMPPCP isolated from their respective NKA crystal structures (top) and in the presence of the proximal binding site residues (middle). It can be seen that the surrounding nonpolar, polar, charged and aromatic residues have minimal impact (<±2 ppm variation) on the calculated chemical shifts as compared to the isolated molecules. The variation in C3′ and C5′ chemical shifts in particular is greater when comparing between ribose N- and S-forms than when comparing between isolated and bound ATP molecules. Hence, the ribose conformation has a greater effect on the calculated chemical shifts than does the binding environment of ATP. Fig. 3a (bottom) depicts the experimental chemical shifts, and errors therein, measured from the spectra in Fig. 2a and summarized in Table 1. After taking into account both the binding environment and ribose conformation, the chemical shifts calculated for ATP in structure PDB 7Y46 all fall within the experimental errors of the measured values. By contrast, many of the shifts calculated for PDB 7WYU and PDB 8D3W fall well outside the experimental errors of the measured values.

Fig. 3b shows correlation plots of the calculated chemical shifts for the bound nucleotides *versus* the experimentally-measured values. The strongest correlation is observed for ATP in the N-form when bound to NKA in the E2.2K^+^ state (PDB 7Y46); the calculated values and experimental values were exceptionally close without applying any correction factors. The error bars for the experimental shifts all overlap with the line of parity between the experimental and calculated values. The correspondence between the calculated shifts for ATP and AMPPCP in the S-form is notably poorer and a first order correction factor is necessary to approach parity between the experimental and calculated values.

Further chemical shift calculations on the binding site clusters extracted from PDB 7WYU and PDB 7Y46 were performed using Gaussian 09 (ref. 47) with PBE and B3LYP functionals (Fig. S4†). The ensembles of ^13^C shift values calculated by this alternative approach again readily distinguished between the N-form ribose of ATP bound to the E2.2K^+^ state and the S-form ribose of ATP bound to the E2P state. The agreement with the experimental data was much closer for calculations using the B3LYP functional than with the PBE functional (Fig. S4b†). As was seen for the plane wave-GIPAW calculations, the shifts calculated for the N-form ribose of ATP bound to E2.2K^+^ were in overall closer agreement with the experimental data (RMSD = 2.9 ppm) than were the shifts for the S-form bound to E2P (RMSD = 6.1 ppm).

**Fig. 4:**
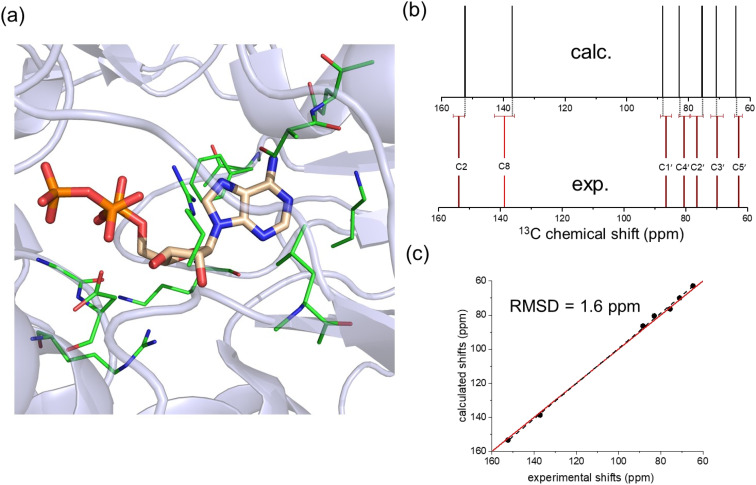
Analysis of a model of ATP with an N-form ribose in the high-affinity nucleotide site of NKA in the E1 conformation. (a) Model of the binding environment. ATP from PDB 7Y46 was docked into the AMPPCP binding site of PDB 8D3W by aligning the adenine bases in Pymol and removing AMPPCP. The coordinates of residues highlighted in green were included with ATP in the chemical shift calculation. (b) Comparison of calculated and experimental ^13^C chemical shifts. (c) Correlation of experimental chemical shifts and calculated values for nucleotide in the presence of binding residues.

The comparison of calculated and measured ^13^C chemical shift values therefore point towards ATP adopting an N-type conformation when bound to NKA in the E1 state. To further strengthen this conclusion, ATP in its ribose N-form conformation bound to E2.2K^+^ was docked into the AMPPCP binding site in the E1 state of the enzyme (Fig. 4a), and chemical shift calculations were performed with CASTEP as before. This was done because the binding environment in the E1 state of the enzyme differs somewhat from that in E2 states. The calculated chemical shifts for the model of ATP in its N-form bound to the E1 state of the enzyme are in excellent agreement with the experimental shifts (Fig. 4b and c).

### SSNMR analysis of the ATP ribose and adenine orientations

The ATP conformation in Fig. 4 is consistent with previous SSNMR measurements of distances between C8 and the phosphate centers in ATP bound to NKA in the E1 state.^4^ The distances measured from the optimized PDB coordinates in Fig. 4 are 4.5 Å for C8–Pα and 4.6 Å for C8–Pβ, which are close to the distances of 4.5 ± 0.5 Å and 4.4 ± 0.4 Å measured by REDOR NMR.^4^ When taken together with the chemical shift analysis, the data suggest that the ATP conformation in the model is close to the actual conformation of ATP in the E1 binding site. A further structural variable is the orientation of the ribose ring relative to the adenine base, which is defined by torsional angle *χ* (C8–N9–C1′–C2′) (Fig. 4a). DFT calculations of the C1′ shift for model nucleic acid molecules have been shown to vary with *χ* in a periodic fashion when the torsional angle is fixed as the only structural variable.^36^ Here, DFT calculations on a cohort of 30 diverse, experimentally-determined ATP structures (from the 272 examined in Fig. 1b) display a periodic dependence (approximated by the first derivative of a Lorentzian function) of the calculated C1′ chemical shift on *χ*. The measured C1′ chemical shift for [U–^13^C]ATP bound to NKA in the E1 state (86.8 ppm) is consistent with a *χ* value in the range −30° to −50°, according to the DFT calculations. The torsional angle of −44° measured from the conformation of ATP in Fig. 4 falls within this range.

**Fig. 5:**
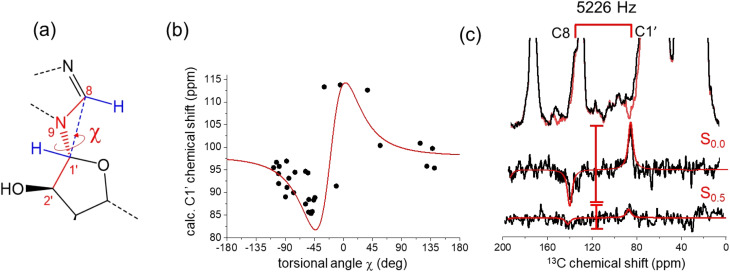
Restraints on the relative orientations of the ribose and adenine rings of ATP bound to NKA, estimated by chemical shifts and by a selective HCCH SSNMR experiment. (a) Torsional angle *χ* defining the relative orientations of the adenine and ribose rings, and its relationship with the C8–H and C1′–H bond orientations. (b) Relationship between *χ* and C1′ chemical shifts calculated from isolated ATP molecules extracted from the PDB structures of 40 proteins, including NKA (7WYU and 7Y46). The red line is the first derivative of a Lorentzian function of width 30° and centered at −20°. The scatter in the data reflects the sensitivity of the C1′ chemical shift to *χ* and to the ribose ring conformation. (c) Experimental ^13^C HCCH SSNMR spectra of the NKA E1·Na^+^ complex with [U–^13^C]ATP obtained with no DQ evolution and after DQ evolution under the local proton field for 0.5*τ*_R_. DQ_0.5_ is given by the ratio of peak difference intensities, *S*_0.5_/*S*_0.0_. Red lines are simulated DQ-filtered peaks based on the ATP geometry in Fig. 4a.

Further evidence that the actual value of *χ* is close to that measured from the ATP conformation in Fig. 4 was obtained with a frequency-selective version of the double quantum (DQ) HCCH SSNMR experiment (Fig. S3†) to determine the relative orientations of adenine C8–H and ribose C1′–H bonds^48,49^ (Fig. 5b and S5, S6;† further details in ESI†). Simulated DQ-filtered peaks, based on the geometry of the ATP conformation in Fig. 4, closely match the experimental data. However, it should be noted that the simulated peaks are also consistent with other geometries (Fig. S6b†). The errors on the measured DQ signal (0.13 ± 0.10; Fig. S6b†) are rather high. This is in part because the DQ efficiency is low because of the relatively large separation of ^13^C nuclear pairs (2.6 Å). In the standard, non-selective HCCH experiment, DQ coherence efficiency is higher between bonded ^13^C pairs, which are typically <1.5 Å apart. The low signal-to-noise arising from the low concentration of ATP (16 nmoles) is also responsible for the large errors. Measurements at higher magnetic field strengths to improve the nuclear spin polarization would not be helpful for this experiment because the higher MAS rates required would reduce the sensitivity of the DQ evolution to the torsional angle. Given these limitations, DNP would be beneficial for the signal-to-noise of the HCCH experiment, and has been shown to enhance the signal from ATP enhancements of around 20-fold in ^13^C and ^31^P.^50^

## Conclusions

Existing atomic structures of NKA indicate that there is no clear relationship between the ATP ribose conformation and the conformation adopted by NKA in the catalytic cycle required for ion pumping.^38–42^ Proteins that use ATP for energy source tend to bind ATP with a C2′-*endo* ribose, whereas proteins that use ATP for phosphorylation tend to bind ATP with a C3′-*endo* ribose.^51^ NKA utilises the energy of ATP hydrolysis and is also phosphorylated, so it is unsurprising that both ribose conformations are adopted. Molecular dynamics simulations may help to better understand the impact of the binding environment and the ribose conformation.

No structural information was hitherto available for authentic ATP when bound to the E1 state of NKA, in part because of the challenges in preventing ATP hydrolysis catalyzed by E1 states of the enzyme. It was possible here to observe ^13^C chemical shifts for all ATP ribose carbons and some adenine carbons for the intact nucleotide when bound to functional NKA in its native membrane and in the E1 state. Rapid freeze-trapping of the enzyme–nucleotide complex protects ATP from hydrolysis during the lengthy NMR measurement times. The availability of this experimental data opened the door to the structural investigation of ATP by chemical shift analysis, exploiting the availability of [U–^13^C]ATP for sensitivity enhancement.

By performing DFT calculations, it is shown here that ^13^C chemical shifts for [U–^13^C]ATP can distinguish between the two main ribose conformations of ATP when bound to NKA. DFT calculations reveal that the ^13^C chemical shifts for the ATP ribose group in the N- and S-forms can differ by over 5 ppm, with the shifts for C3′ and C5′ having the highest sensitivity to conformation. The sensitivity of ^13^C chemical shift values to the ribose conformation of isolated ATP and other nucleotides has been reported before, but it was not known at the outset of this work how the shift values would be influenced by interactions with amino acid residues in the NKA nucleotide binding sites. A key finding of this work is that the ATP binding environment has a minor impact on the ribose ^13^C chemical shifts, compared to the effect of the ribose ring conformation. Comparison of the calculated and experimental shift values points in favour of an ATP ribose moiety bound to the E1 state of NKA that closely resembles ATP bound to the E2:2K^+^ state and is in the N-(C3′-*endo*) conformation. One caveat is that water molecules were not included or approximated in the calculations. However, inspection of the highest-resolution crystal structures of NKA indicates that water molecules are excluded from the high-affinity nucleotide site.

The DFT method employed here is the plane wave-GIPAW approach, which is normally applied to calculations of periodic systems. Although this approach is also suitable for calculations on single molecules and atomic clusters in large simulation boxes, GIAO calculations using Gaussian may be considered superior for non-periodic systems in terms of computational economy and accuracy. The reduced computational time of the latter allows for the implementation of more accurate exchange-correlation density functionals (*e.g.*, hybrid functionals like B3LYP) higher up the “Jacob's ladder” of approximations. However, when considering the uncertainties of the measured chemical shifts imposed by the experimental peak widths (Table 1), the plane wave approach with GGA was found to be sufficiently accurate to answer the specific question asked here, namely, to distinguish between the N- and S-form of the ATP ribose in the nucleotide site. In cases where the precision of the experimental shift data is higher than seen here, calculations using alternative DFT methods would be advisable.

The majority of DFT computational time was consumed by geometry optimisations, which each took on the order of days compared to just a few hours for the magnetic shielding calculations on the optimised molecule. Full-atom optimisation was used for consistency throughout here, but considering that the heavy atom coordinates were derived from experimental measurement, optimisation of only the hydrogen positions would be more computationally economical and potentially lead to more accurate shift calculations. Chemical shift calculations were performed only on optimised systems for which the RMSD of the atomic coordinates was within the resolution of the structure from which they were extracted. For the highest resolution structures (<1.7 Å), hydrogen optimisation was subsequently found to be sufficient, whereas for the lowest resolutions (>4 Å), the full atom approach is justifiable.

In summary, we have demonstrated that chemical shifts can be utilized to report on the conformation of an organic ligand when bound to a membrane-embedded protein.

## Conflicts of interest

There are no conflicts to declare.

## Supplementary Material

RA-013-D3RA06236H-s001
