# Supplementary material for: Solid-state NMR chemical shift analysis for determining the conformation of ATP bound to Na,K-ATPase in its native membrane
Source: RSC Adv. 2023 Nov 29;13(49):34836–46. doi: 10.1039/d3ra06236h (PMC10685339; doi:10.1039/d3ra06236h)
Supplement: RA-013-D3RA06236H-s001 [file RA-013-D3RA06236H-s001.pdf]

Electronic Supplementary Material (ESI) for RSC Advances.
This journal is © The Royal Society of Chemistry 2023

Supporting Information for:

Solid-state NMR ^{13}C chemical shift analysis of the ATP ribose ring conformation and orientation when bound to a protein embedded in its native membrane

David A. Middleton, John Griffin, Mikael Esmann, Natalya U. Fedosova

Supplementary Information: the selective HCCH experiment

The usual broadband HCCH experiments on uniformly ^{13}C molecules excite double quantum (DQ) coherences between bonded pairs of carbons to measure HCCH torsional angles sequentially (Figure S3a). Here, a selective HCCH experiment (Figure S3b) was used to excite only the ^{13}C DQ coherence between non-bonded C8 and C1' by adjusting the MAS frequency to the n = 1 rotational resonance condition, at which the MAS frequency is set to the difference in the chemical shifts of the two carbons (5226 Hz at an applied magnetic field B_0 of 9.3 T). The DQ coherence is measured after evolution for a period t of up to one cycle of sample rotation, t_R, under the influence of the local dipolar field of the bonded protons, H8 and H1'. The evolution of DQ coherence, measured as a difference intensity S(t), is sensitive to the effective torsional angle, H8 – C8 – C1' – H1', and the effective bond angle C8 – C1' – H1', both of which vary according to χ (Figure S4). S(t) is also dependent on the effective bond angle C1' – C8 – H8, which does not vary with χ and is taken to be 126°. Ideally, a series of S(t) values are measured from t = 0 s to $t = t_R$ and the evolution compared with simulated curves to obtain values of χ. However, the sensitivity of the HCCH experiment is very low and each spectrum required acquisition of 230,000 transients with block averaging to achieve suitable signal-to-noise. Therefore only two spectra were obtained, at t = 0 and at $t = 0.5t_R$ to obtain the normalized value of $S_{0.5}$ (defined in Figure S5a), which is shown to vary with the value of χ according to Figure 5b and is highest for positive values of χ. Figure 3c of the main text shows HCCH NMR spectra at t = 0 and at $t = 0.5t_R$ overlaid with simulated peaks scaled according to Figure 5a. A value of $S_{0.5} = 0.12 \pm 0.10$ was determined, the uncertainty reflecting the signal-to-noise.

Supplementary Figures

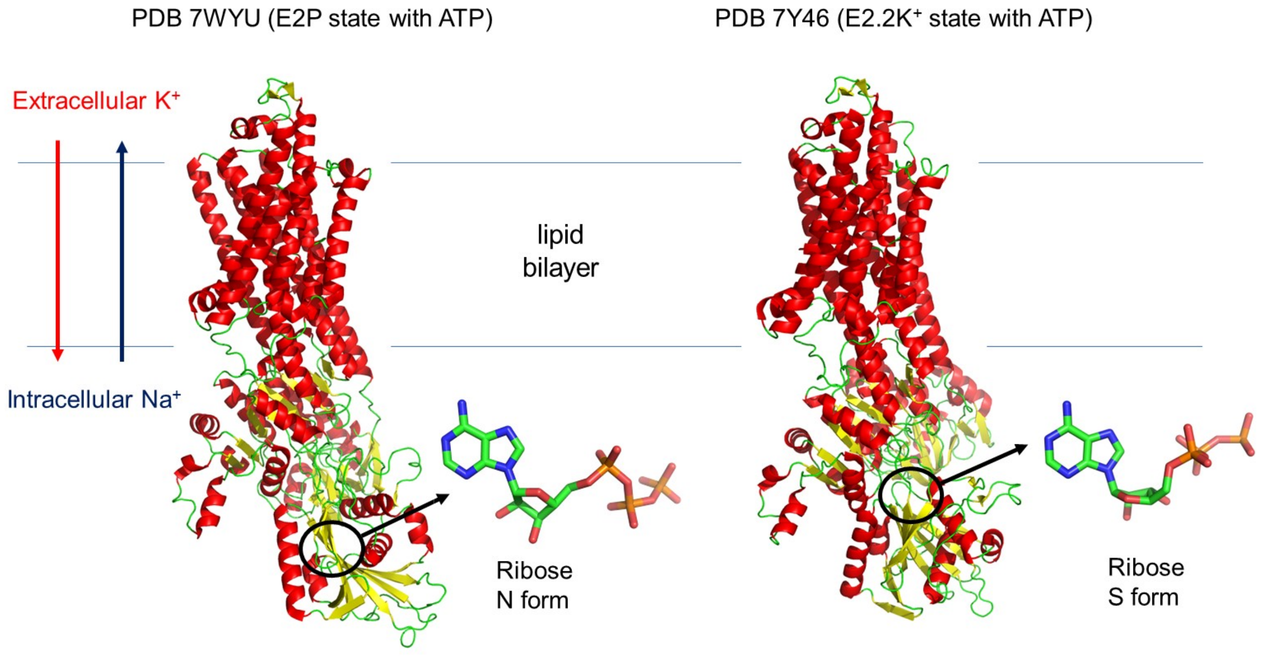

Figure S1. Two representative structures of the NKA α-subunit in E2 conformations complexed with ATP.

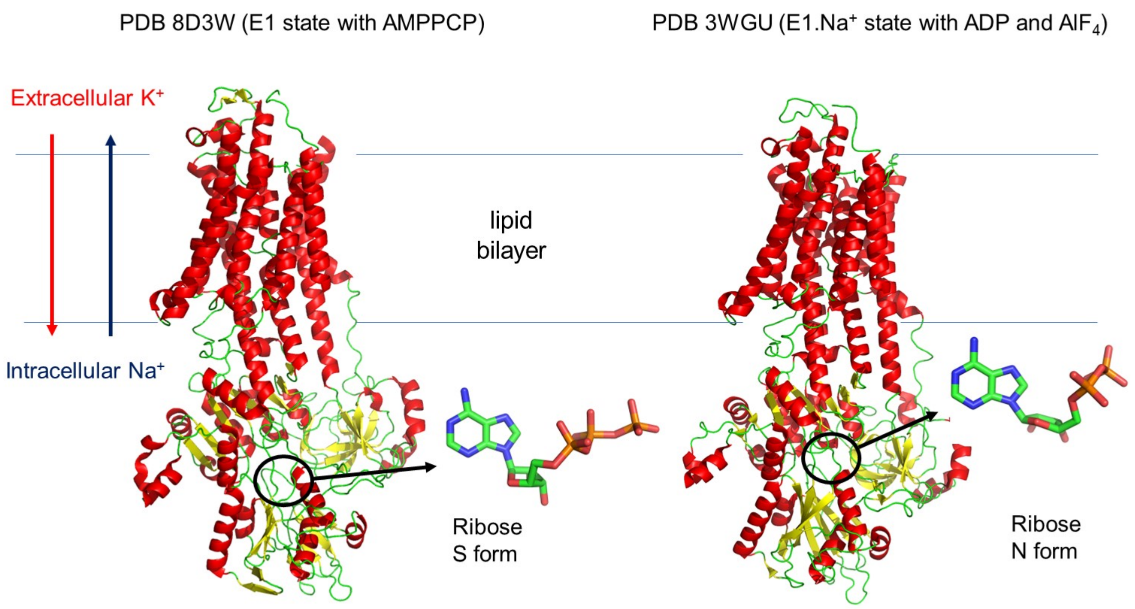

Figure S2. Two structures of the NKA α-subunit in E1 conformations complexed with non-hydrolysable ATP analogues.

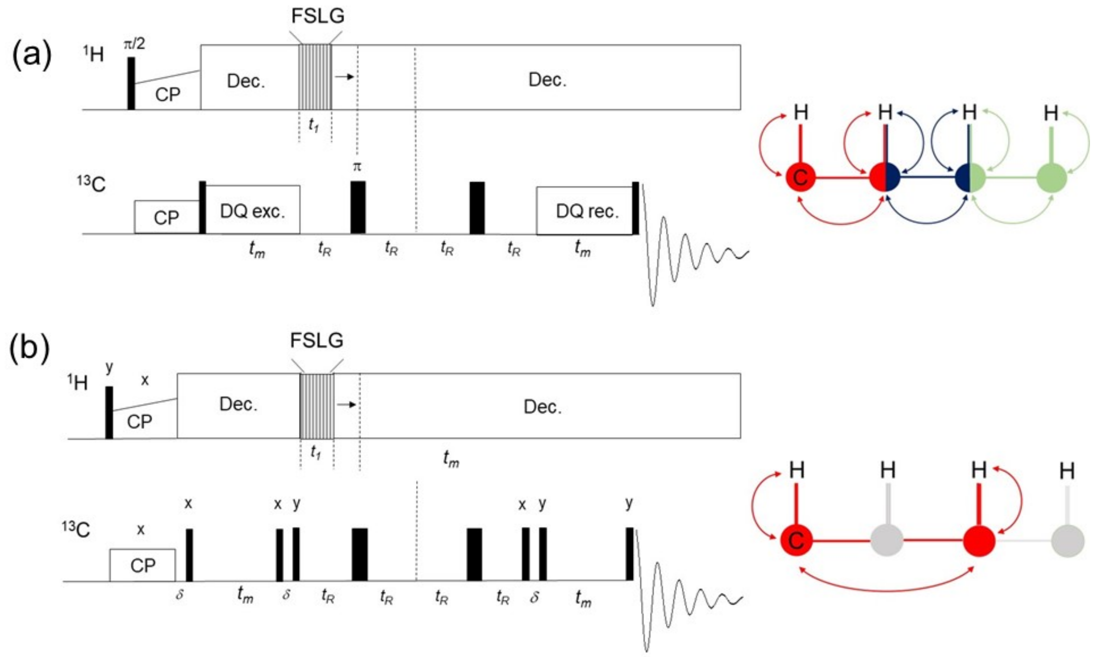

Figure S3. Two HCCH SSNMR pulse sequences. (a) HCCH with broadband excitation of DQ coherence, as described elsewhere. This experiment typically excites DQ coherences between directly bonded pairs of carbons in a uniformly ^{13}C labeled molecule, which evolves under frequency-switched Lee-Goldberg (FSLG) proton homonuclear decoupling for one rotor period before conversion back into observable magnetization. It is usually run as a two-dimensional experiment to resolve individual DQ coherences. (b) The HCCH with selective excitation of DQ coherence at rotational resonance. This experiment can in principle excite DQ coherence between any pair of carbons in a uniformly ^{13}C labeled molecule, depending on the difference in their chemical shifts, which is matched to the MAS frequency. This experiment was used to determine the torsional angle χ.

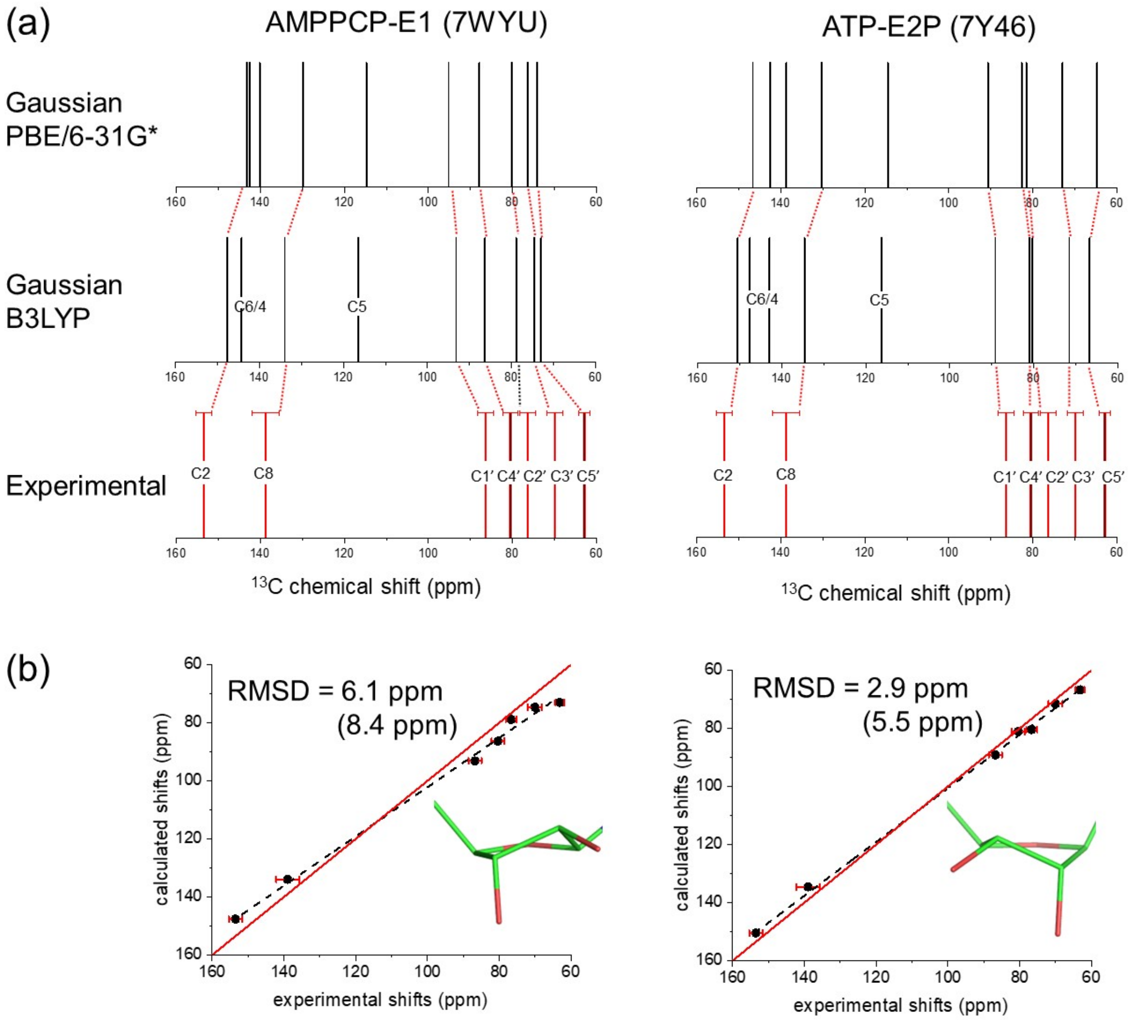

Figure S4. Comparison of experimental ^{13}C chemical shifts with values calculated with Gaussian using PBE and B3LYP functionals, for AMPPCP bound to NKA in the E1 conformation (left) and in the E2P conformation (right). (a) Calculated shift values. (b) Correlation of experimental values and values calculated with the B3LYP hybrid functional. RMSD values are given for the B3LYP calculations and PBE calculations (in brackets). All calculated values are based on the same binding site clusters taken from PDB 7WYU and PDB 7Y46 as shown in Figure 2b of the main text.

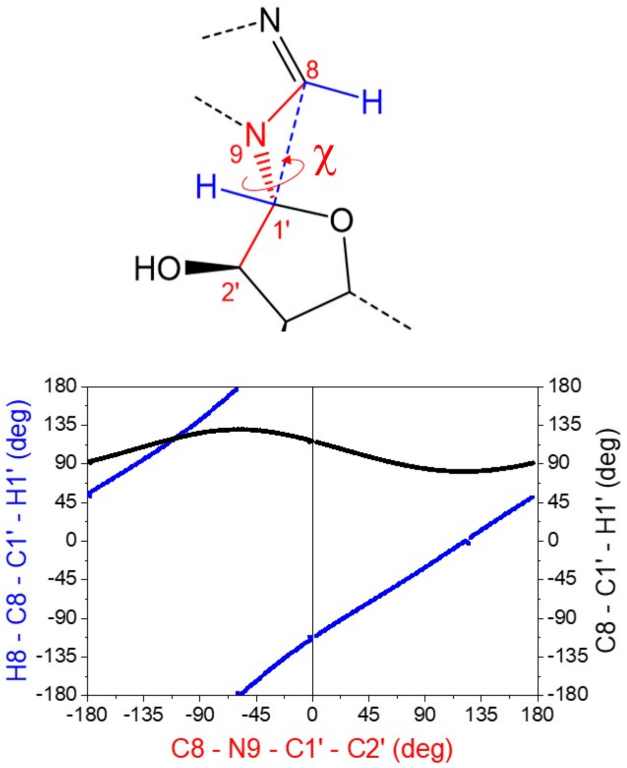

Figure S5. Relationship between angle χ (C8 – N9 – C1’ – C2’) and the effective torsional angle H8 – C8 – C1’ – H1’ and effective bond angle C8 – C1’ – H1’ of ATP as the ribose group is rotated about the N9 – C1’ bond. Values are based on the actual bond lengths and angles of ATP represented in Figure 4a.

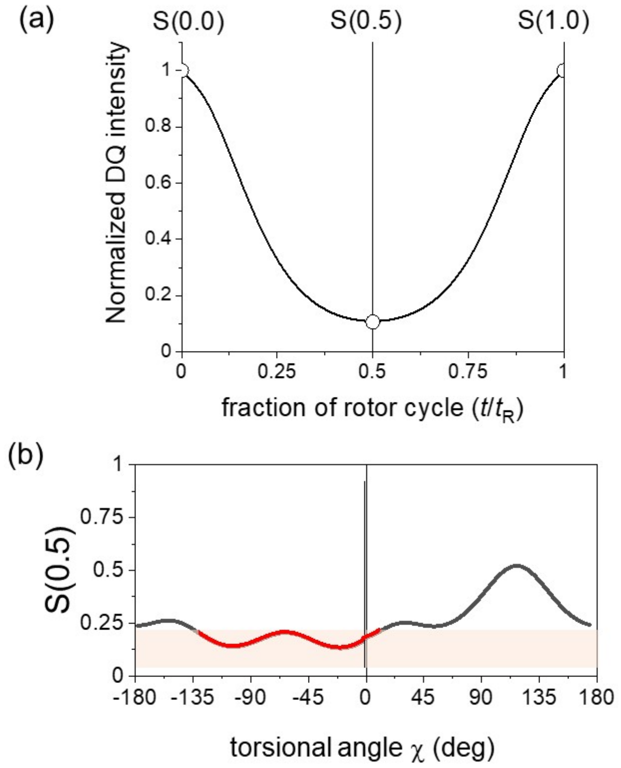

Figure S6. Evolution of DQ coherence in the selective HCCH NMR experiment. (a) DQ evolution over one cycle of sample rotation at the magic angle, calculated according to the molecular geometry of ATP in Figure 4a of the main text. The DQ intensity is the difference in the integrals measured from the resonances for C8 and C1', normalized to the value at t = 0 s. The values of S(0.0) and S(0.5) were used to calculate the simulated peak intensities in Figure 5c of the main text. Input values are: the effective torsional angle H8 – C8 – C1' – H1' (164°), effective bond angle C8 – C1' – H1' (129°) and effective bond angle H8 – C8 – C1' (96°). These values apply to a torsional angle χ of -44°. The dipolar coupling between bonded ^{1}H – ^{13}C pairs was calculated to be -21300 kHz, based on an average internuclear separation of 1.12 Å, and a scaling factor κ of 0.45 was applied. The value of κ was obtained by calibration with a crystalline leucine sample of known geometry. A MAS frequency of 5226 Hz, corresponding to n = 1 rotational resonance with respect to C8 and C1' was assumed. (b) Variation of S(0.5) as a function of χ. The shaded region represents a measured value of S(0.5) of 0.13 ± 0.10. The red portion of the continuous line represents the values of χ that are consistent with the measured value of S(0.5).
